# Exploring the Parallel G-Quadruplex Nucleic Acid World: A Spectroscopic and Computational Investigation on the Binding of the c-myc Oncogene NHE III1 Region by the Phytochemical Polydatin

**DOI:** 10.3390/molecules27092997

**Published:** 2022-05-07

**Authors:** Francesca Greco, Domenica Musumeci, Nicola Borbone, Andrea Patrizia Falanga, Stefano D’Errico, Monica Terracciano, Ilaria Piccialli, Giovanni Nicola Roviello, Giorgia Oliviero

**Affiliations:** 1 Department of Pharmacy, University of Naples Federico II, Via Domenico Montesano 49, 80131 Naples, Italy; francesca.greco@unina.it (F.G.); nicola.borbone@unina.it (N.B.); andreapatrizia.falanga@unina.it (A.P.F.); stefano.derrico@unina.it (S.D.); monica.terracciano@unina.it (M.T.); 2 Institute of Biostructures and Bioimaging, Italian National Council for Research (IBB-CNR), Area di Ricerca Site and Headquarters-Via Pietro Castellino 111, 80131 Naples, Italy; domenica.musumeci@unina.it; 3 Department of Chemistry, University of Naples Federico II, Via Vicinale Cupa Cintia 21, 80126 Naples, Italy; 4 ISBE-IT, University of Naples Federico II, 80138 Naples, Italy; 5 Division of Pharmacology, Department of Neuroscience, Reproductive and Odontostomatological Sciences, University of Naples Federico II, Via Sergio Pansini 5, 80131 Naples, Italy; ilaria.piccialli@unina.it; 6 Department of Molecular Medicine and Medical Biotechnologies, University of Naples Federico II, Via Sergio Pansini 5, 80131 Naples, Italy; golivier@unina.it

**Keywords:** Pu22, G-quadruplex, c-myc, phytochemicals, circular dichroism, in silico simulations, molecular docking, CD prediction

## Abstract

Trans-polydatin (tPD), the 3-β-D-glucoside of the well-known nutraceutical trans-resveratrol, is a natural polyphenol with documented anti-cancer, anti-inflammatory, cardioprotective, and immunoregulatory effects. Considering the anticancer activity of tPD, in this work, we aimed to explore the binding properties of this natural compound with the G-quadruplex (G4) structure formed by the Pu22 [d(TGAGGGTGGGTAGGGTGGGTAA)] DNA sequence by exploiting CD spectroscopy and molecular docking simulations. Pu22 is a mutated and shorter analog of the G4-forming sequence known as Pu27 located in the promoter of the c-myc oncogene, whose overexpression triggers the metabolic changes responsible for cancer cells transformation. The binding of tPD with the parallel Pu22 G4 was confirmed by CD spectroscopy, which showed significant changes in the CD spectrum of the DNA and a slight thermal stabilization of the G4 structure. To gain a deeper insight into the structural features of the tPD-Pu22 complex, we performed an in silico molecular docking study, which indicated that the interaction of tPD with Pu22 G4 may involve partial end-stacking to the terminal G-quartet and H-bonding interactions between the sugar moiety of the ligand and deoxynucleotides not included in the G-tetrads. Finally, we compared the experimental CD profiles of Pu22 G4 with the corresponding theoretical output obtained using DichroCalc, a web-based server normally used for the prediction of proteins’ CD spectra starting from their “.pdb” file. The results indicated a good agreement between the predicted and the experimental CD spectra in terms of the spectral bands’ profile even if with a slight bathochromic shift in the positive band, suggesting the utility of this predictive tool for G4 DNA CD investigations.

## 1. Introduction

Among the noncanonical secondary structures of DNA, G-quadruplex (G4) is an appealing therapeutic target being found in specific regions of the genome such as telomeres and the regulatory regions of many oncogenes including c-kit, c-myc, and bcl-2 [1,2,3,4,5,6,7,8,9,10,11,12,13,14]. Remarkably, the promoter region of c-myc—an oncogene over-expressed in the majority of solid tumors and closely associated with cancer cell apoptosis, proliferation, invasion, cell-cycle arrest, and metastasis—can form a parallel G4 structure via Hoogsteen hydrogen bonds under specific conditions, and has been proposed as an effective target for antitumor drugs [15,16,17,18,19,20,21,22,23]. Particularly, it was found that molecules capable of binding and stabilizing this type of G4 downregulate the expression of c-myc, finally resulting in the apoptosis of cancer cells with great benefit in anticancer therapy [24,25,26].

**Figure 1:**
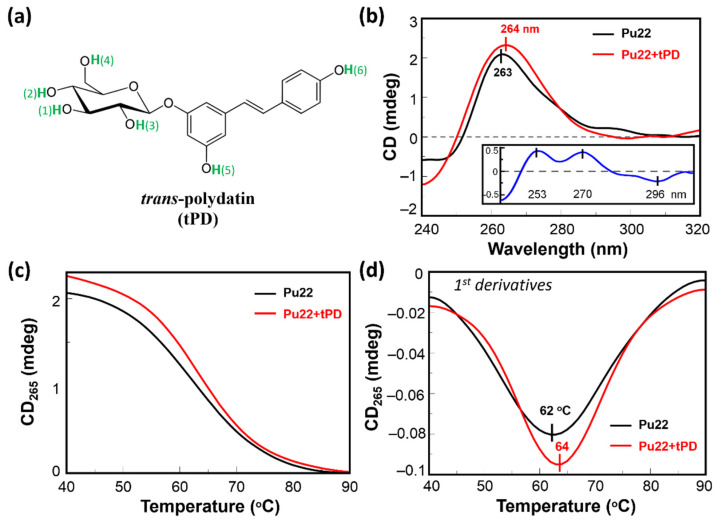
(**a**) Chemical structure of tPD; some atoms are numbered as in the docking program. (**b**) CD spectra of Pu22 2.5 μM (black) and its complex with tPD (red) at 40 °C. Inset shows the “difference” CD spectrum (tPD-Pu22 (mdeg)–Pu22 (mdeg)). (**c**) CD thermal denaturation curves (CD_265_ (mdeg) vs. T (°C)) and (**d**) their first derivatives vs. T plots for Pu22 (2.5 μM, black) and its complex with tPD (red). All experiments were run in PBS, pH 7.4 (optical path length = 0.1 cm).

Trans-polydatin (tPD, Figure 1a), the 3-β-D-glucoside of the well-known nutraceutical trans-resveratrol [27], is a natural polyphenol with documented anti-cancer, anti-inflammatory, cardioprotective, and immunoregulatory effects [28,29]. In a recent work, the G4-binding of tPD was explored toward three cancer-related G-rich DNA sequences, including c-myc, in comparison with a model duplex [30]. Interestingly, tPD displayed a clear binding ability with all the G4s and a higher ability, with respect to its aglycone derivative trans-resveratrol, to discriminate G4 over duplex DNA. Moreover, in vitro assays on melanoma cells proved that tPD significantly reduced telomerase activity, and inhibited cancer cell proliferation [30]. However, the adopted experimental conditions did not allow the detection of any significant conformational changes of the analyzed G4 DNA upon binding with tPD. Moreover, it was not possible to estimate the thermal stability of both c-myc and its complex with tPD, as needed for evaluating any stabilizing or destabilizing effects of the polyphenol on the G4-folded c-myc promoter [30]. On the other hand, other studies clearly indicated that the anticancer effects (including inhibition of cell proliferation and metastasis) of tPD took place through suppressing the c-myc expression, as proven in a model of human cervical cancer [31]. Therefore, conscious of the role of G4-structure binding and stabilization by ligands in c-myc deregulation [32], we decided to examine in more detail the molecular recognition of c-myc G4 by tPD through an approach differing from that previously reported in the literature from both experimental and in silico perspectives. To this aim, the interaction of tPD with c-myc DNA was studied in the present work focusing on the G4 structure formed by the Pu22 region having the sequence 5′-TGAGGGTGGGTAGGGTGGGTAA-3′, a mutated and shorter analog of the sequence known as Pu27 located in the promoter of the c-myc oncogene and associated with the regulation of promoter activity and gene transcription.

Circular dichroism (CD) spectra of Pu22, either unliganded or in complex with the tPD, were recorded at variable temperatures in a buffer containing a lower concentration of potassium ions than previously reported [30]. CD spectroscopy is a technique typically employed to verify the formation of several secondary structures of nucleic acids and their analogs [33,34,35,36], including the G4 structure in G-rich DNA sequences [37,38,39,40,41], which also allows one to determine whether the denaturing temperature of a DNA secondary structure is affected by potential ligands [42]. Being aware of the utility of molecular docking in identifying DNA ligands [43,44] through verification of the favored binding sites in a complex, and in the estimation of the binding affinity, we decided to further characterize the molecular recognition of the G4 by tPD, by docking experiments of the tested polyphenol against the c-myc G4. The CD spectrum of the parallel G4 structure of Pu22 was further predicted by DichroCalc software [45], with the aim to verify whether the experimental profile could be reproduced by simulation as described below.

## 2. Results

The effective binding of polydatin to Pu22 had been unequivocally shown by some of us by using various techniques including fluorescence [30]; however, with our work, we aimed to explore some biophysical characteristics of the complex, such as its thermal stability, and give more insights into the molecular aspects of the interaction by using in silico approaches. Our combined experimental and computational work started with the examination of the CD spectral features of Pu22 DNA and its complex with tPD. Moreover, a thermal denaturation study was conducted with both unliganded Pu22 and tPD-Pu22. The observations from CD spectroscopy were then interpreted in the light of the docking experiments performed by us on tPD-Pu22, but also on (tPD-Pu22)-Pu22, (Pu22)_2_, and tPD-(Pu22)_2_ molecular systems.

### 2.1. CD Spectroscopic Analysis of the Binding of Pu22 by tPD

With the aim to shed light on the possible mechanisms underlying the previously reported anticancer activity of tPD [31], we evaluated the potential of this polyphenol in binding Pu22. In our CD study, we observed a spectrum for Pu22 corresponding to a G4 with parallel topology, as identified by the characteristic positive band at ~265 nm and the negative one at 240 nm (Figure 1b, black line) [46]. In the presence of the polyphenol, we observed an increase in the positive CD signal at 263 nm accompanied with a 1 nm red-shift in the band maximum, and a concomitant reduction in the CD minimum at 240 nm (Figure 1b, red line). In addition, some differences in the CD spectra were evidenced in the 280–300 nm region. Overall, in the studied conditions, tPD induced a greater degree of structuration in the Pu22 G-quadruplex, as evidenced by the “difference” CD spectrum obtained by subtracting the CD spectrum of the Pu22 G4 to that of the tPD-Pu22 complex (inset of Figure 1b).

**Figure 2:**
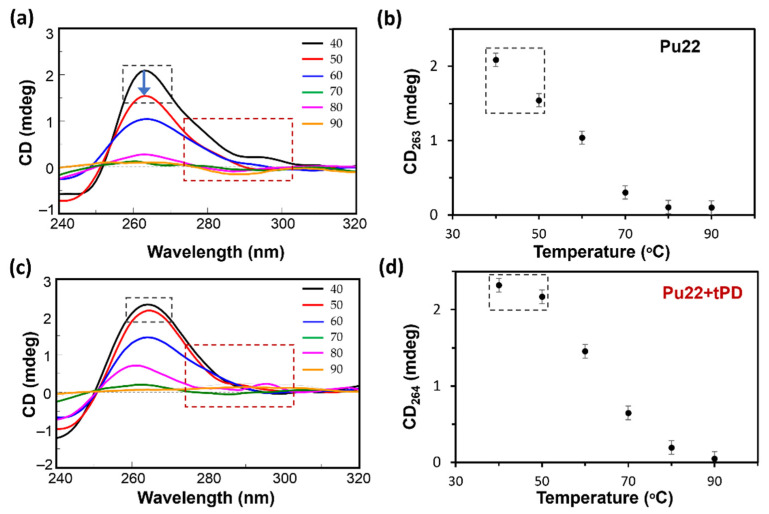
CD spectra of Pu22 (2.5 μM) (**a**) and its complex with tPD (**c**) recorded in the 40–90 °C temperature range. Plots of the CD signal at λ_max_ (in mdeg) vs. temperature (in °C) for Pu22 (**b**) and its complex with tPD (**d**) derived from panels **a** and **c**. All experiments were run in PBS, pH 7.4 (optical path length = 0.1 cm).

Then, we studied the effect of tPD on the stability of this G4 DNA by recording, for Pu22 and its mixture with the polyphenol, the CD values at 265 nm as a function of temperature (Figure 1c). We found a slight thermal stabilization in the presence of tPD, detectable by the increased value of the G4 melting temperature (T_m_ = 64 °C) with respect to the unliganded Pu22 G4 reference (T_m_ = 62 °C), leading to a ΔT_m_ of +2 °C (Figure 1d and Table 1). Furthermore, the overall variation in the CD signal at the λ_max_ upon heating, i.e., between 40 (folded state) and 90 °C (unfolded), for Pu22 alone or in complex with tPD, was 1.99 and 2.23, respectively, again confirming a higher structuration degree of the quadruplex when bound by the ligand. Specifically, the highest difference in the ΔCD for the two systems was evidenced between 40 and 50 °C (Figure 2, black-line dashed squares, and Table 1). Some differences between the two systems were also detected in the CD spectra recorded at the various temperatures in the 280–300 nm spectral region (Figure 2a,c).

### 2.2. Molecular Docking Studies

**Figure 3:**
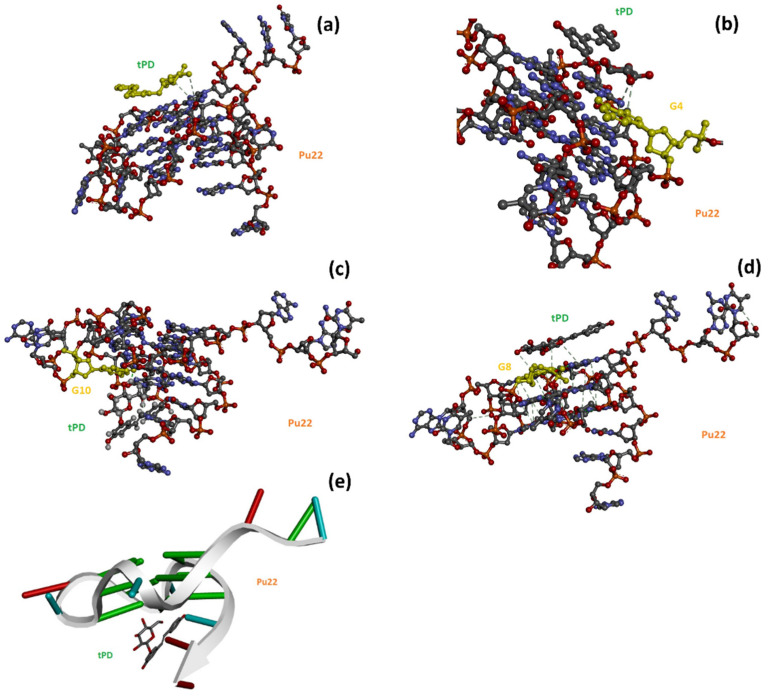
The docked structures of the tPD-Pu22, with the Pu22 PDB ID: 6AU4, corresponding to the top-1–3 ranked poses: (**a**,**b**) pose 1; (**c**) pose 2; (**d**) pose 3. Note how in poses 1 and 3, tPD seems to interact by end-stacking and H-bondings with the nucleotides represented in yellow in panels (**b**,**d**). Panel (**e**) reports a different depiction of pose 2 in which the backbone of Pu22 is represented as a white arrow and the base pairs as ladders for clarity.

The importance of phytochemicals in drug discovery [47] prompted the scientific community to investigate the potential of a plethora of natural products in anticancer strategies by using in silico approaches for a rapid screening or to corroborate and describe at a molecular level in vitro observations. In this context, we used herein in silico methods, and more specifically molecular docking, in analogy to other recent literature examples using polyphenols as anticancer drug candidates [48,49], to deeper analyze the interaction between tPD and the target Pu22 G4, whose sequence is located in a regulatory region of the c-myc oncogene. More in detail, we exploited the Hdock software [50,51] for the computational studies involving DNA. Hdock is used for both macromolecule–macromolecule [50] and small molecule–macromolecule [52] dockings, including those involving DNA and RNA G4s [53,54]. It is worth noting that the docking software provides dimensionless scores (Hdock scores) that are correlated to binding affinities [55]. This allows the comparison to made of the binding affinity of ligands for a given target by simply comparing their docking scores, with the most negative values being associated with the highest affinity ligands [55]. We found by Hdock docking that tPD bound the G4 target in proximity of the G4, G8, G13, and G17 nucleotides (Figure 3, Table 2). Comparing the Hdock scores for the top-1 poses, we can predict that the ligand bound Pu22 with a lower affinity than its aglycone resveratrol (tRES, Table 2), as experimentally shown in the literature [30].

The interactions that emerged by analyzing the top-1–3 poses are held by H-bondings with aromatic rings involving the tPD H1 (Figure 1a) and the guanine residues 4 (3.15 Å, ligand H1–G-ring; π donor H-bond) and 8 (3.05 Å, ligand H1–G ring; π donor H-bond), respectively, in poses 1 and 3 (Figure 3b,d). In pose 2, a H-bond between ligand H2 (Figure 1a) and the O6 (2.14 Å) of guanine residue 10 was also detected (Figure 3c). Interestingly, unlike pose 2 (Figure 3c,e), poses 1 and 3 show the tPD aromatic moieties laying almost parallel to the terminal quartets of the quadruplex (Figure 3), thus suggesting a partial end-stacking interaction of the polyphenol to the G4.

The dimerization of Pu22 G4 was described in the literature under some conditions; for example, a quadruplex dimer was clearly evidenced in the solid state [56], whereas in solution, this G4 is present mainly as a monomer [57]. Nonetheless, Jana and Weisz [58] using nondenaturing polyacrylamide gel electrophoresis showed that in solution, *MYC*-Δ1,6 and, albeit to a much lesser extent, Pu22 (indicated by them as “*MYC*-Δ1,6[1.2.1]”, carrying two G replacements by T with respect to *MYC*-Δ1,6) presented dimeric forms corresponding to slower migrating bands, more evident in the former case and somewhat faint, but still detectable, in the case of Pu22 [58]. Similarly, the electrophoretic assays of Moriyama et al. showed for Pu22 (indicated by them as c-myc) a main band and two slower migrating bands [59]. The presence of dimeric Pu22 in solution was suggested also by size exclusion chromatography (SEC), revealing two main SEC peaks for the Pu22 solution that led to the hypothesis of the coexistence of monomeric and dimeric forms in solution [60]. Therefore, we hypothesize that Pu22 in solution is found mainly as a monomer, which justifies its usage in biomolecular studies as a model of G4 DNA not prone to undesirable multimerization, but also, albeit at a much lesser degree, as a dimer. G4 DNA dimer binding by ligands could, in principle, alter the monomer-dimer equilibrium, and importantly, some ligands can induce dimerization of truncated parallel c-myc G-quadruplexes [61].

With all the above considerations in mind, we decided to explore by molecular docking also the propensity of tPD to bind the (Pu22)_2_ dimer model. We found for the top-ranked pose, as well as poses 1–3 of this docking, less negative Hdock scores (−103.2 and −102.7 ± 0.5, respectively; Table 2) with respect to those found in the case of the docking of the same ligand with the monomeric G4 (−112.1 and −111.7 ± 0.3), suggesting a slightly higher affinity of tPD for the most abundant monomeric form of the Pu22 G4 structure.

**Figure 4:**
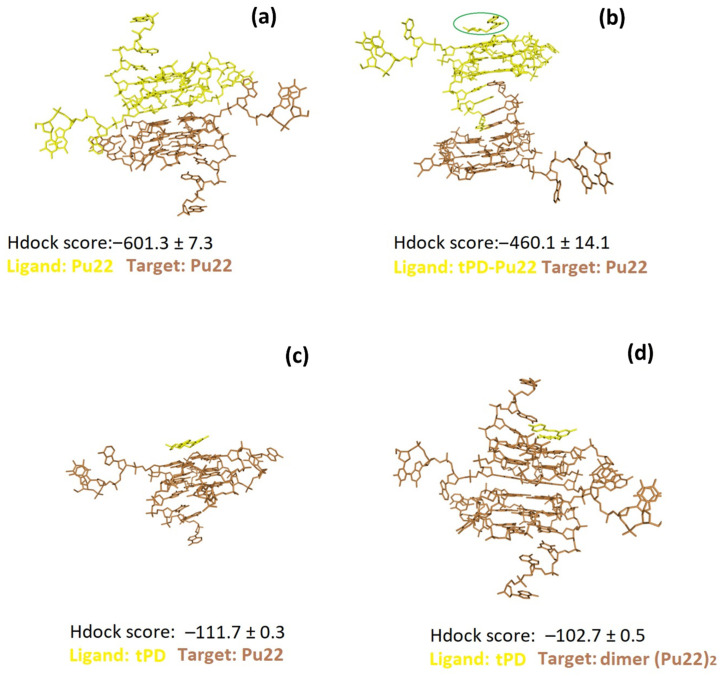
Docking of Pu22 (**a**) or tPD-Pu22 (**b**) to another Pu22 unit. Docking of tPD to Pu22 monomer (**c**) or dimer (**d**). Hdock scores (mean of top-1–3 values) were also indicated.

We also performed DNA–DNA dockings to explore the dimerization of Pu22 G-quadruplex to obtain (Pu22)_2_ and the effects of tPD on this process. To this scope, in the first case, we docked Pu22 G4 to a second Pu22 G4 unit, set as the target (Figure 4a), while in the second docking, we used the pre-docked tPD-Pu22 G4 for docking to a second Pu22 G4 unit (Figure 4b). We found that tPD-Pu22 G4 binds Pu22 G4 with an affinity 1.3 times lower than that showed by unliganded Pu22 G4 with the same target (Hdock scores (mean of top-1–3 values): −460.1 ± 14.1 vs. −601.3 ± 7.3, respectively). In other terms, it seems that tPD hinders Pu22 G4 dimerization that, in its absence, is more favored (Figure 4a,b), and binds the Pu22 G4 monomer with slightly higher affinity than the dimeric (Pu22)_2_ G4 (Figure 4b,d).

**Figure 5:**
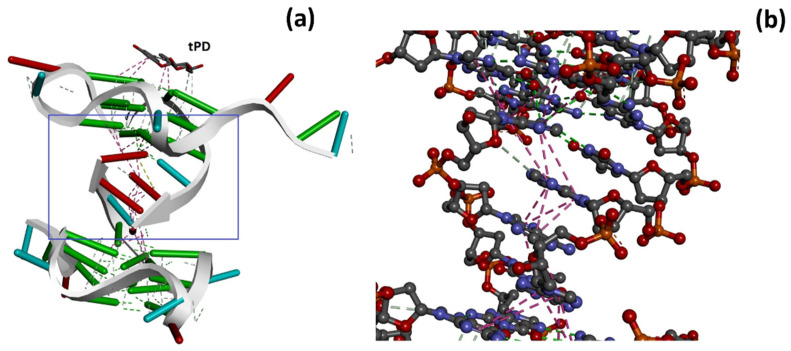
(**a**) Detailed pose view of the trimeric complex (tPD-Pu22)-Pu22 of Figure 4b; tPD structure is indicated. (**b**) Enlargement of the area delimited by the blue rectangle.

Remarkably, the dimeric form of Pu22 G4 with tPD (Figure 4b and Figure 5a) was predicted to show considerable structural differences with respect to the unliganded (Pu22)_2_ G4 dimer (Figure 4a). In this regard, it is worth noting how 18 hydrophobic/π–π stacking Pu22-Pu22 intermolecular interactions (pink, Figure 5b) along with six intermolecular H-bonds (green) are predicted to sustain the trimeric complex structure.

### 2.3. CD Predictions and Comparison with Experimental Spectroscopic Data

**Figure 6:**
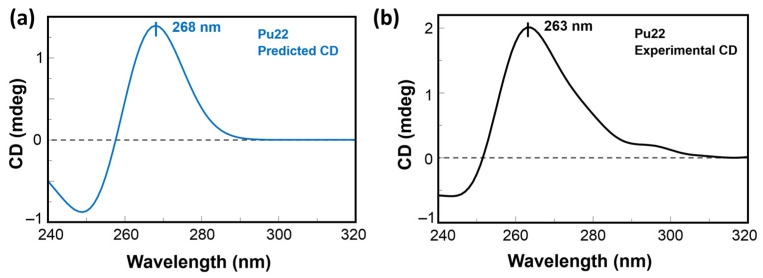
Theoretical CD spectrum of Pu22 G4 (**a**) as simulated by DichroCalc [45] using the PDB ID 1XAV, compared with the experimental counterpart (**b**) obtained for Pu22 at 2.5 μM in PBS.

Furthermore, the solution NMR structure of the monomeric model of Pu22 G4 formed in human c-myc promoter [57] was used to simulate its CD spectrum by DichroCalc [45]. This software is routinely used for obtaining simulated CD spectra of proteins starting from their PDB structure files. In our approach, we applied the method to the prediction of the spectroscopic profile of the G4 structure object of our study. In particular, a positive band at 268 nm and a negative one at 244 were predicted by DichroCalc (Figure 6a), which were, to some extent, in analogy to what we experimentally found by CD (Figure 6b) and was previously described in the literature for the parallel G4 structure of Pu22, though with a bathochromic shift in the bands by about 5 nm.

## 3. Discussion

With this investigation, we aimed to give more insights into the interaction of the natural polyphenol tPD with the G4-forming DNA model of the c-myc promoter Pu22, as the anticancer effects of this phytochemical compound were previously associated to c-myc deregulation [31]. Specifically, a possible mechanism of anticancer activity could be the stabilization of a G4 structure within a regulatory region of this oncogene. Previous attempts [30] in this regard failed to show any stabilization effects of tPD due to the experimental conditions used and notably because of the particularly K^+^-rich buffer [30]. In this work, we decided to substitute the previously used buffer with PBS, which corresponds to an overall 4.5 mM K^+^ concentration. The binding of tPD with the parallel Pu22 G4 was confirmed by CD spectroscopy, which showed changes in the CD spectrum of this DNA secondary structure under our experimental conditions, especially in the characteristic positive band centered at 263 nm (Figure 1b). The overall variation in the CD spectrum of Pu22 when bound by tPD was significant and evidenced by the “difference” CD spectrum (inset of Figure 1b). The thermal denaturation profiles in PBS revealed for both Pu22 and tPD-Pu22 sigmoidal shapes with transition midpoint temperatures (T_m_s) of 62 and 64 °C, respectively (Figure 1c,d, Table 1), denoting a stabilization effect of tPD on the G4. Furthermore, by examining the variations in the CD curves upon heating, we observed that tPD in the complex slowed down the unfolding process of the G4 structure, especially in the 40–50 °C range. Then, to give a tentative interpretation of the experimental findings, we conducted a molecular docking study on different systems including Pu22 monomeric and dimeric G4s and tPD. The most interesting docking results revealed that tPD may bind the monomeric G4 c-myc model (PDB ID: 6AU4 [56]) used in our CD experiments by partial stacking to the terminal G-quartet of the 22-mer sequence Pu22 (Figure 3). The binding involves a region similar to that described previously [30] for the 24-mer G4 structure (PDB ID: 2A5P), in the proximity of nucleotides common to both computational studies, such as G13 [30] (Table 2). The tPD-Pu22 complex is held also by H-bonding interactions with the aromatic rings [62] between the tPD hydrogen H1 and the guanines in positions 4 and 8. There is also an H-bond between ligand H2 and the O6 of guanine 10 (pose 2), but we cannot exclude that other intermolecular forces (for instance, hydrophobic interactions) contribute to the complex formation. Interestingly, the tPD aromatic moieties in two poses out of three lay almost parallel to the quartets of G4 (Figure 3), thus suggesting a partial π–π stacking of the polyphenol to the terminal G4 quartet (end-stacking). In our hypothesis, the partial end-stacking of tPD to the G-quartet could have a role in the experimental CD thermal behavior observed, as this interaction could reinforce the G4 stabilizing it. Our docking suggests that polydatin could bind the G4 structure by end-stacking as reported in the literature for other stilbene derivatives [63]. Interestingly, Esaki et al. [64] found that naphthalene derivatives are able to stack with the quadruplex G-quartet and afforded thermal stabilizations similar to those observed by us with polydatin, which are also comparable to those recorded for polydatin and resveratrol with the G4s tel26 and hTERT [30]. When tPD is bound to Pu22, it leads to the formation of a complex in which 18 hydrophobic/π–π stacking intermolecular interactions along with six intermolecular H-bonds sustain a trimeric structure of polydatin-(Pu22)_2_, although it prevents Pu22 G4 dimerization with a full eight-floor coplanar system (Figure 4a). In this regard, other ligands of G-quadruplex DNAs were able to induce dimerization in monomeric G4-forming sequences, such as truncated c-myc promoter DNAs [61], leading also to G4 thermal stabilization [65].

## 4. Materials and Methods

### 4.1. Materials

All the reagents and solvents were of the highest commercially available quality and were used as received from Sigma-Aldrich (Merck S.r.l., Milan, Italy). Pu22 DNA sequence d[TGAGGGTGGGTAGGGTGGGTAA], purchased by Eurofins (Turin, Italy) in lyophilized and desalted form, was dissolved in nuclease-free bidistilled water and quantified by UV measurements of the absorbance at 260 nm at 95 °C using as extinction molar coefficient of 228,700 M^−1^ cm^−1^ (ssDNA, nn model, https://atdbio.com/tools/oligo-calculator, accessed on 3 March 2022). The DNA stock solution had a 200 µM concentration. Stock solutions of tPD ligand (kind gift of Prof. G Ravagnan) were prepared at 8 mM concentration in DMSO.

### 4.2. CD Studies

Circular dichroism (CD) spectra were registered with procedures similar to previous literature reports [66] on a Jasco J-810 (Jasco Europe S.R.L., Cremella, Italy) spectropolarimeter, equipped with a Peltier ETC-505 T temperature controller, in a Hellma (Milan, Italy) quartz cell with a light path of 0.1 cm. The spectra were recorded within the 240–320 nm wavelength range and corrected by subtracting the contribution of the solvents. All experiments were performed in PBS buffer (137 mM NaCl, 2.7 mM KCl, 10 mM Na_2_HPO_4_, and 1.8 mM KH_2_PO4, pH 7.4; Sigma Aldrich, Milan, Italy), using 2.5 µM DNA (Pu22), diluted from the stock solution in water, and 125 µM tPD (50 equiv. respect to the DNA), diluted from the stock solution in DMSO.

### 4.3. CD Denaturation Studies

All G4-containing solutions were annealed by heating them at 95 °C for 5 min and then letting them slowly cool down to room temperature (over 16 h). The presented melting curves (obtained by recording CD_265nm_ vs. T in the 40–90 °C temperature range) are representative of three independent experiments. Melting temperature (T_m_) values were determined as the temperatures relative to the minima of the 1st derivative plots of the denaturation curves. All experiments were repeated at least three times and all spectra were recorded in triplicate.

### 4.4. Molecular Docking

We conducted our blind molecular docking with the program HDOCK [50,51] using default parameters for all dockings and the PDB entry 6AU4 that is suitable for studies involving dimerization (selecting one of the two G4 monomers) of the Pu22 G4 structure [56]. The HDOCK server uses the iterative knowledge-based scoring function ITScore-PP to rank the top-ten poses provided after each docking run. The HDOCK score furnished by the program is an energy score whose values are listed as dimensionless, and larger negative numbers of the HDOCK score indicate stronger binding interactions between the interacting ligand/macromolecules, which was reported to correlate well to experimental binding affinities.

We used the 3D structure of the Pu22 DNA with the PDB (Protein Data Bank) ID: 6AU4 [56]. The 3D structure, including H-atoms, for the natural compound trans-polydatin, was retrieved by us from the PubChem database (https://pubchem.ncbi.nlm.nih.gov/, accessed on 8 November 2021). More details on the HDOCK docking server and on the procedures for docking experiments can be found at http://hdock.phys.hust.edu.cn/ (accessed on 9 November 2021). We analyzed the top-ranked pose (Top-1) and the top-three ranked poses for the complexes predicted by HDOCK according to the energy scores provided by the program as explained in the Results section. Ligand/DNA complexes were visualized by Discovery Studio 2021 software (Accelrys, San Diego, CA, USA) [67] that was used also for analyzing H-bonding between tPD and G4 DNA.

### 4.5. CD Predictions

The prediction of the CD spectrum of the monomeric Pu22 G4 structure was performed using the DichroCalc [45] web server starting from the PDB file of the NMR structure deposited with PDB ID 1XAV. At first, the 1XAV.pdb file was manually edited by replacing the unrecognized “DA, DC, DG, DT” text for deoxyribonucleotides with “A, C, G, T”. Then, the edited PDB file was uploaded as the input file in DichroCalc obtaining the predicted CD spectrum file, which was edited with SpectraGryph 1.2 [68]. The predicted CD spectrum from the “ds” format was finally visualized in Jasco Spectra Manager (JASCO Corporation, Sendai, Japan).

## 5. Conclusions

Here, we described a combined approach including in silico (molecular docking) and experimental (CD binding assay/CD thermal denaturation) analyses, through which we verified that tPD can interact with Pu22, a G4-forming sequence related to the promoter region of the c-myc oncogene, stabilizing this DNA structure. The tPD anticancer activity previously observed in vitro correlates with its stabilizing effects on this cancer-related target. The interaction of tPD with the parallel quadruplex has been proven by CD, showing changes in the CD spectrum of this DNA secondary structure under our experimental conditions, especially in the characteristic positive band centered at 263 nm. Moreover, slight thermal stabilization effects on the G4 by tPD have been revealed by CD melting studies. The binding with the DNA structure has been described in more detail in silico by molecular docking, which suggests that the interaction of tPD with Pu22 G4 may take place through partial end-stacking to the terminal quartet involving deoxynucleotides placed in the external regions of the G4 and the sugar moiety of the ligand. Finally, the exploitation of the DichroCalc web-based server, normally used for the prediction of CD spectra of proteins, for the computation of CD spectra of Pu22 revealed the feasibility of the method for the predictions of CD spectra of G4 DNA.

## Figures and Tables

**Table 1:** Summary of the CD and CD melting data for Pu22 and the complex tPD-Pu22. ΔT_m_ is the variation in the melting temperature of the complex with respect to the Pu22 reference; ΔCD_max 40–90_ is the difference in the CD value at the λ_max_ between 40 (folded state) and 90 °C (unfolded), whereas ΔCD_max 40–50_ is the one between 40 and 50 °C.

Entry	ΔT_m_ * (°C)	ΔCD_max 40–90 °C_ (mdeg)	ΔCD_max 40–50 °C_ (mdeg)
Pu22	0	1.99	0.54
tPD-Pu22	+2	2.23	0.15

* T_m_ Pu22 = 62 °C.

**Table 2:** HDOCK docking scores (for the top-ranked pose and mean value from the top-1–3 poses). The interface nucleotide residues within 5.0 Å from the ligand in the top-1–3 complexes are reported in the last column.

Complex	HDOCK Score * Top-1 Ranked Pose	HDOCK Score Mean Value (Top-1–3 Poses) ± SD	Interface Residues
tPD/Pu22	–112.1	–111.7 ± 0.3	G4, G6, G8, G10, G13, G15, T16, G17, G19, T20, A21
tRES/Pu22	–120.6	–112.9 ± 7.3	G6, T7, G10, G15, T16, G19, T20, A21
tPD/(Pu22)_2_	–103.2	–102.7 ± 0.5	G6, G10, T11, G15, T16, G19, T20, A21, G’14, G’15, T’16, G’17, G’18, G’19, T’20, A’21

* The docking energy scores.

## Data Availability

Not applicable.
